# Modern treatment of perineuriomas: a case-series and systematic review

**DOI:** 10.1186/s12883-020-01637-z

**Published:** 2020-02-13

**Authors:** Anne-Kathrin Uerschels, Christos Krogias, Andreas Junker, Ulrich Sure, Karsten H. Wrede, Oliver Gembruch

**Affiliations:** 1 Department of Neurosurgery, University Hospital Essen, University of Duisburg-Essen, Essen, Germany; 2 Department of Neurology, St. Josef-Hospital Bochum, Ruhr University Bochum, Bochum, Germany; 3 Department of Neuropathology, University Hospital Essen, University of Duisburg-Essen, Essen, Germany

**Keywords:** Intraneural perineurioma, Surgical treatment, Target fascicular biopsy, Decompression and neurolysis

## Abstract

**Background:**

Perineuriomas are rare benign peripheral nerve sheath tumours of perineurial cell origin and can be classified into intraneural and extraneural perineuriomas. They most commonly present a mononeuropathy of gradual onset and slow progression, resulting in progressive neurological deficits like hypoesthesia or motor weakness. Therapy is still variable. Aim of the study was to compare our surgical treatment and our follow-up regime including high-resolution nerve sonography with the current literature to evaluate best treatment of perineuriomas.

**Methods:**

Retrospective analysis of our dataset “peripheral nerve lesion” to identify patients suffering from perineuriomas between 01.01.2012 until 31.12.2018. Surgical treatment and the follow-up examination of three patients were described. Additionally, a systematic review including PubMed, the Cochrane Collaboration Library, Scopus and Google Scholar was performed for literature published between January 1, 1990 and October 31, 2019 independently by 2 authors.

**Results:**

In the first case, the left ulnar nerve was affected. In the second case, the left peroneal nerve and in the third case the right median nerve was affected. High-resolution nerve sonography was performed in each case. All patients underwent interfascicular neurolysis combined with a targeted fascicular biopsy under electrophysiological monitoring. Neurological deficits improved subsidized by rehabilitation. Surgical therapy and the neurological outcome were compared with literature. Systematic review revealed 22 articles, which met the inclusion criteria. Therefore, demographics, surgical treatment and neurological outcome of 77 patients were analysed.

**Conclusions:**

Perineuriomas are rare benign nerve sheath tumours with a slow progression, sometimes difficult to diagnose. Decompression and neurolysis may improve neurological deficits. High resolution nerve sonography might serve as a helpful additional diagnostic tool in this process.

## Background

Perineuriomas (PN) are rare benign peripheral nerve sheath tumours originating from perineurial cells. Intraneural and extraneural PN have to be distinguished. The intraneural PN is restricted to the boundaries of the peripheral nerve, whereas the extraneural PN is found mainly in soft tissues and skin [1].

They arise from perineural cells located in the perineurium of the peripheral nerve, which is histologically characterized by a complex perineurial cell proliferation, which extends into the endoneurium and surrounds concentrically individual myelinated and the unmyelinated axon-Schwann cell complexes of peripheral nerve fascicles and endoneurial capillaries. This leads to the characteristic “pseudo-onion bulbs” [2]. It is known that the perineurium forms the blood-nerve barrier and has continuity with the pia-arachnoid membrane of the central nervous system [3, 4]. A neoplastic origin for intraneural and soft tissue PN is suggested due to the presence of 22q deletions [5, 6]. However, recently performed genomic analysis indicates divergent pathogenetic mechanisms of intraneural PN and soft tissue PN. The intraneural PN frequently contains TRAF7 mutations [7], and rarely, chr22q12 deletions whereas the soft tissue PN showed no TRAF7 mutations. Alterations in NF1 or NF2 likely contribute to perineurioma pathogenesis, similar to other nerve sheath tumours [8].

They most commonly present a mononeuropathy of gradual onset and slow progression, resulting in progressive neurological deficits like hypoesthesia or motor weakness [9, 10]. It is believed that the motoric fascicles are affected by the tumour, there as the sensory deficits are caused by the intraneural compression of the sensoric fascicles.

Those lesions can be difficult to diagnose due to their rarity. Up to now, guidelines do not exist, and the management of this rare pathology is still various.

Therefore, we retrospectively analysed our dataset “peripheral nerve lesion” to identify patients suffering from perineuriomas. Aim of the study was to compare our surgical treatment and our follow-up regime including high-resolution nerve sonography (HRNS) with the current literature.

## Methods

### Patients

Retrospective analysis of our dataset “peripheral nerve lesion” was performed between 01.01.2012 until 31.12.2018. Three patients suffering from an intraneural PN were identified. Patients were treated in our department using following surgical technique: microsurgical interfascicular neurolysis in combination with a biopsy of a non-functional fascicle. Medical records, pre- and postoperative HRNS and MRI were analysed. Follow-up examination and postoperative HRNS were evaluated up to 2½ years after surgery.

The study was approved by the Institutional Review Board (Medical Faculty, University of Duisburg-Essen, Registration number: 18–7955-BO).

### Surgery

Preoperative HRNS was used to identify the affected nerve and to plan the extend of the skin incision followed by sterile washing and draping of the surgical field. After skin incision, careful preparation towards the nerve was performed. The affected nerve can be identified by its pathologically changed surface structure with an enlarged epineurium that covers the thickened fascicles. Intraoperative HRNS was now used to visualise the affected nerve and to plan the extend of the epineural opening. Microsurgical interfascicular neurolysis can be achieved after careful longitudinal incision of the epineurium. Electrophysiological stimulation (0.1–0.2 mA) of each fascicle shows the ones with motor function. An enlarged fascicle without motor function is identified for a targeted fascicular biopsy (4 mm length). If possible, end-to-end anastomosis should be performed afterwards to prevent microneuroma.

### Systematic review of the literature

A systematic search via PubMed, the Cochrane Collaboration Library, Scopus and Google Scholar was performed for literature published between January 1, 1990 and October 31, 2019 independently by 2 authors.

Search key words comprised “intraneural perineurioma”, “intraneural perineuriomas” and “localized hypertrophic mononeuropathy”. Inclusion criteria were articles published in English presenting the clinical course, peripheral nerve location, and the treatment regime. Therefore, cases without description of the surgical treatment were excluded.

**Fig. 1:**
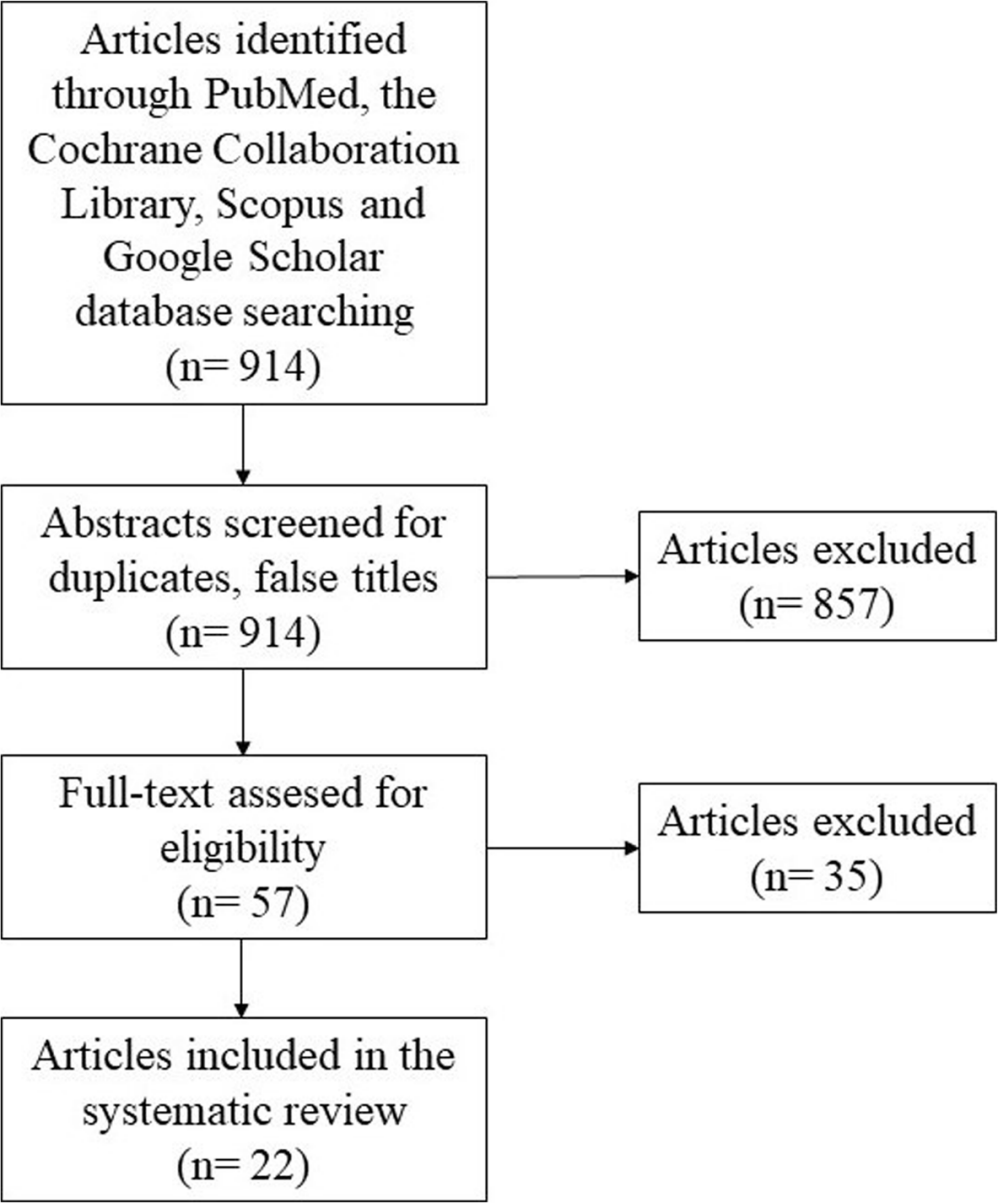
PRISMA flow chart demonstrating the systematic review of the literature

Afterwards, the reference lists of included articles were reviewed to identify and include additional eligible articles. Furthermore, all included studies were meticulously cross-referenced to ensure that patients were not included in multiple articles (Fig. 1). The systematic review was conducted following *Preferred Reporting Items for Systematic Reviews and Meta-Analyses* guidelines [11].

### Statistical analysis

Data were analysed using SPSS 23.0 (Statistical Package for the Social Sciences, SPSS Inc., Chicago, IL, USA). Metric data were described by mean and standard deviation and nominal data by frequency and valid percent.

## Results

### Case presentations

#### Case 1

**Fig. 2:**
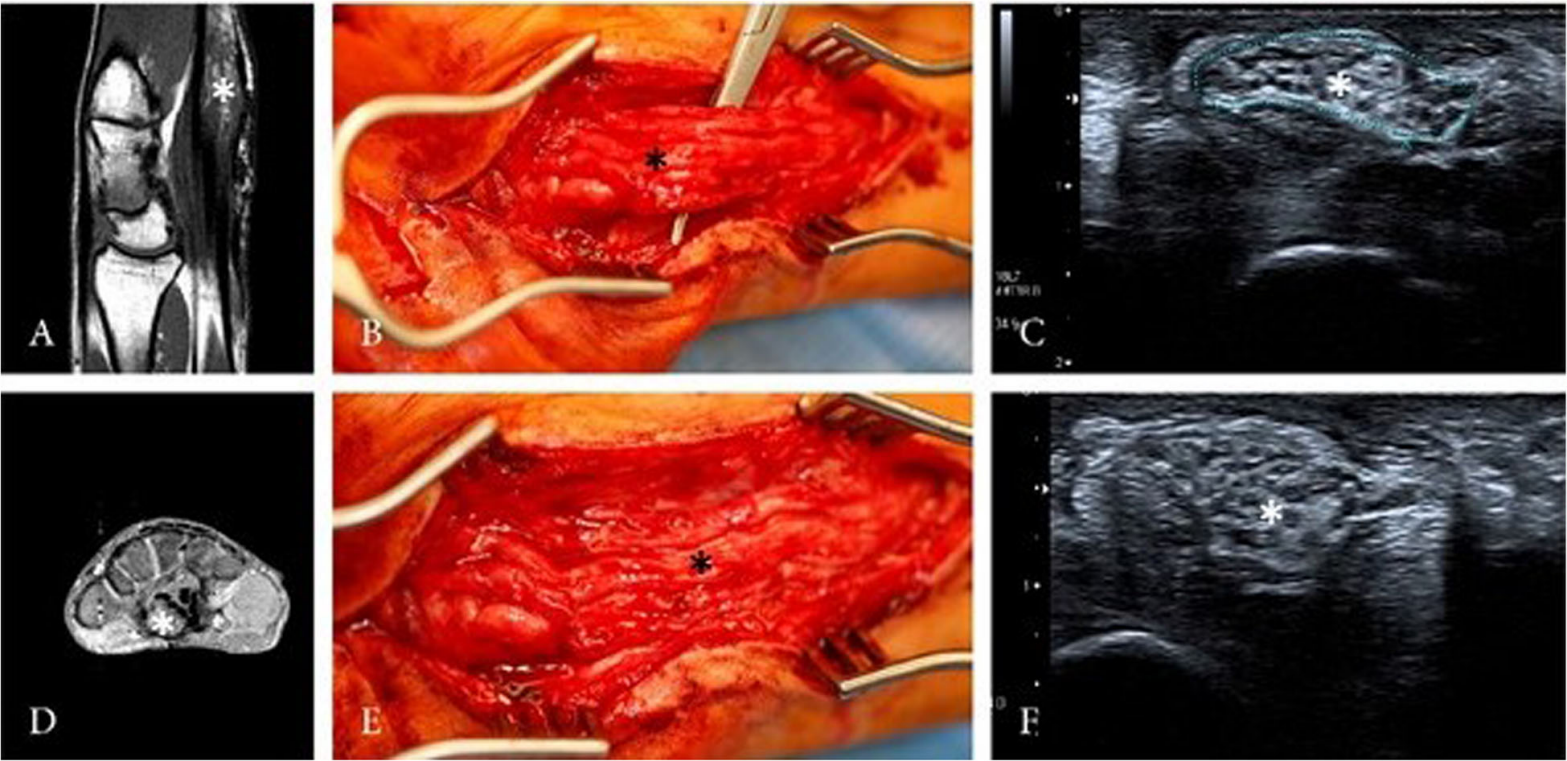
HRNS (**c** + **f**) and MRI (**a** + **d**) of the right-hand depicted nerve enlargement (asterisk) with perineurial tissue and still identifiable fascicular structure. Intraoperative view showing the perineurioma (**b** + **e**)

A 56-year-old male patient with typical clinical and electroneurographic findings of carpal-tunnel-syndrome caused by an intraneural PN of the right median nerve presented to our neurosurgical department. The patient complaint about a progressive hypaesthesia and paresis of the hand and underwent 4 surgeries over a period of 30 years without long term benefit because of suspected carpal-tunnel-syndrome. HRNS and magnetic resonance imaging (MRI) of the right-hand depicted an intraneural PN of the median nerve (Fig. 2). Follow-up examination 27 months after surgery showed an improvement in motor function undergoing physiotherapy, but no change of the sensory deficits. HRNS revealed stable intraneural PN.

#### Case 2

**Fig. 3:**
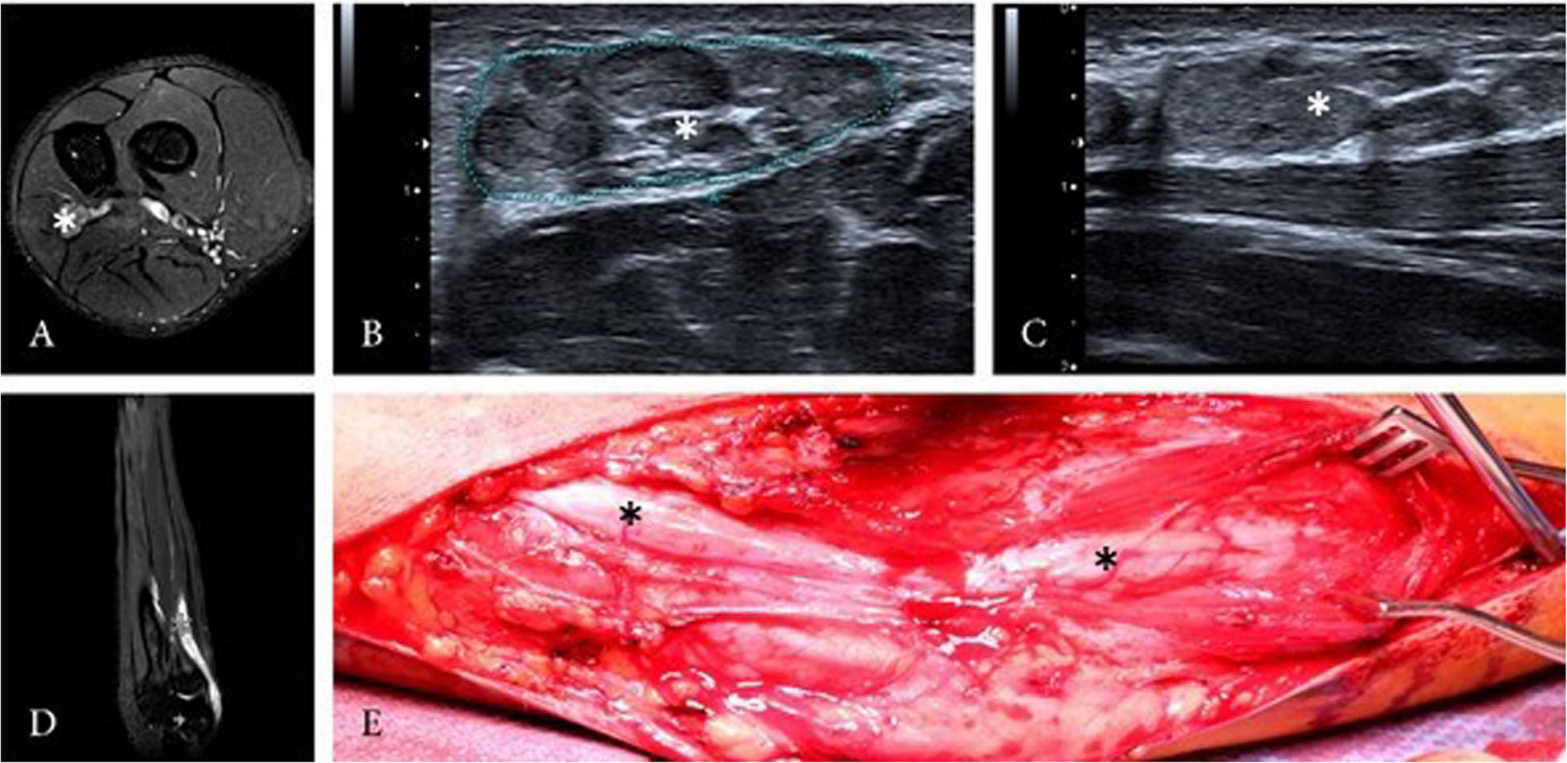
HRNS (**b** + **c**) and MRI (**a** + **d**) of the left forearm depicted nerve enlargement (asterisk) with perineurial tissue and still identifiable fascicular structure. Intraoperative view showing the perineurioma (**e**)

A 16-year-old left-handed male patient presented with an intraneural PN of the left ulnar nerve. Symptoms lasted over 2 years, showing a progressive atrophy of the intrinsic muscles of the left hand without any sensory deficit. Electrophysiological testing revealed an impairment of the left ulnar nerve, HRNS and MRI of the left arm showed intraneural PN of the ulnar nerve with a length of about 15 cm reaching from the middle of the upper arm to the middle of the forearm (Fig. 3). Despite the atrophy of the intrinsic muscles of the hand, follow-up examination 24 months after surgery showed a stable state of the PN on HRNS, without any sensory deficit. The functionality of the hand improved under consequent physiotherapy.

#### Case 3

**Fig. 4:**
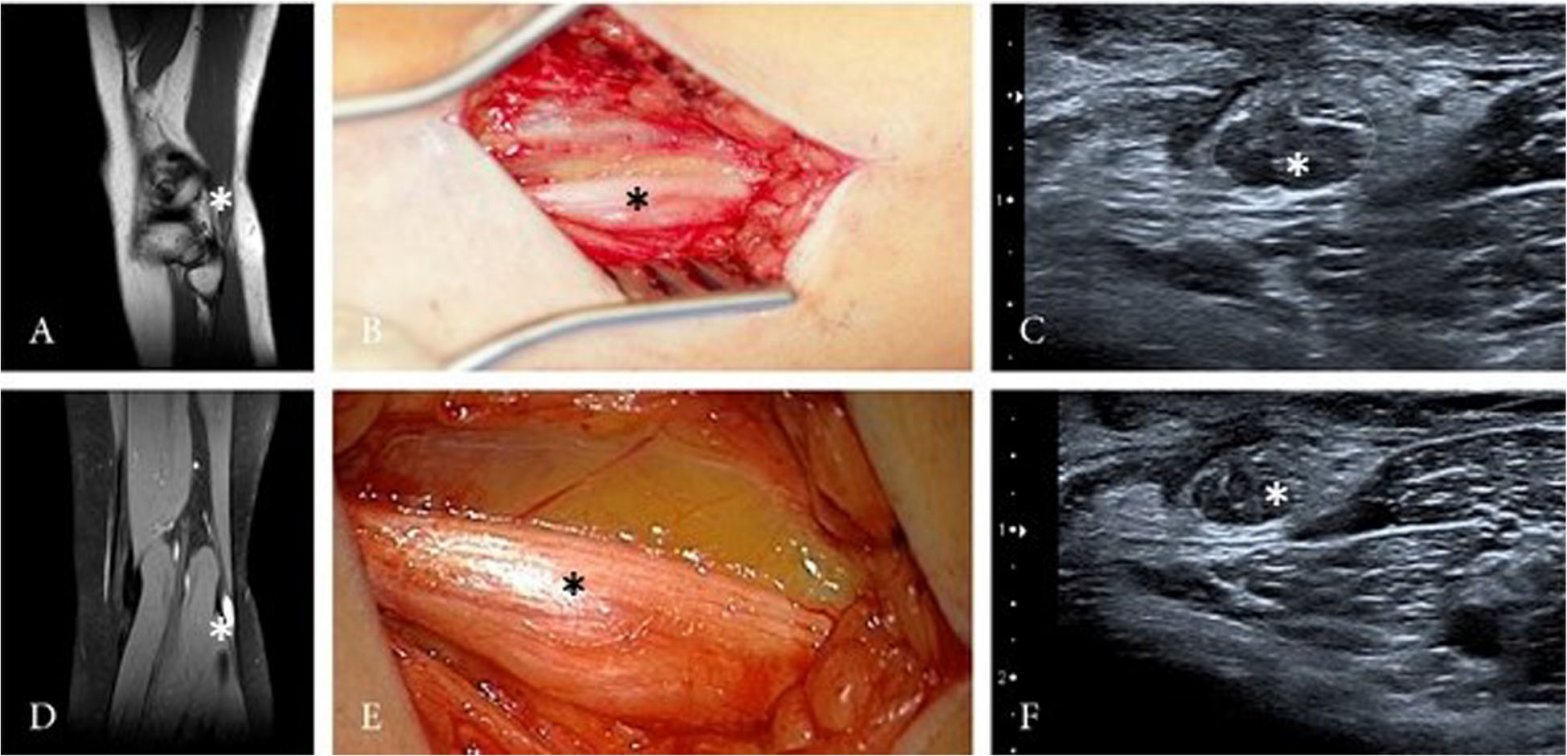
HRNS (**c** + **f**) and MRI (**a** + **d**) of the left upper limb depicted nerve enlargement (asterisk) with perineurial tissue and still identifiable fascicular structure. Intraoperative view showing the perineurioma (**b** + **e**)

A 17-year-old female patient complained about progressive impairment of foot elevation over a period of 3 years, showing a paresis of 2/5. The patient was treated by the neurologist due to the suspicious of an idiopathic peroneal paresis. Electrophysiological testing showed impairment of the left peroneal nerve. HRNS and MRI of the left leg revealed an intraneural PN of peroneal nerve (Fig. 4). Postoperative, the paresis of the foot elevation increased to 1/5. Follow-up examination 30 months after surgery showed an improvement of the foot elevation, now 3/5.

### Histopathology

**Fig. 5:**
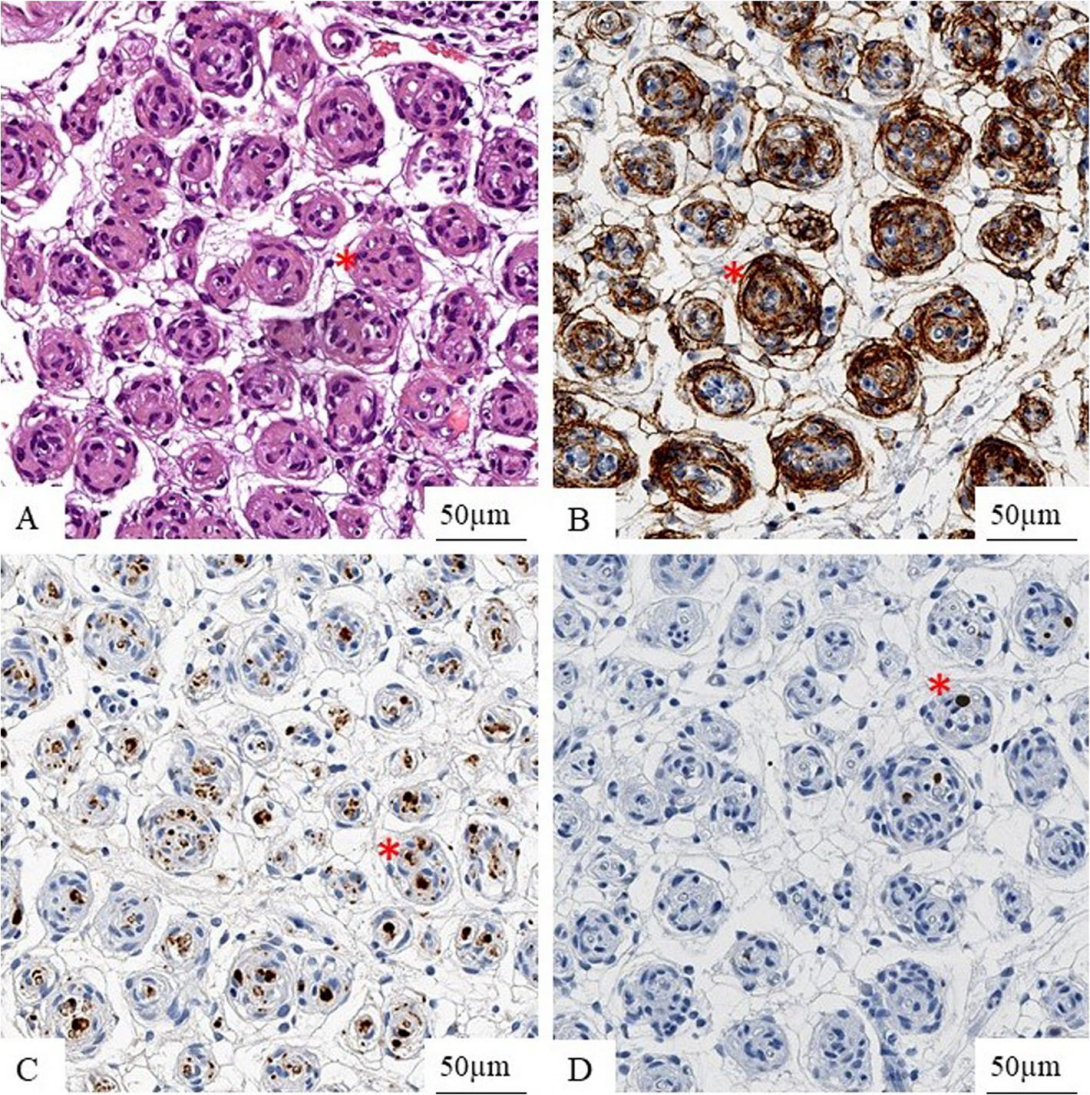
Histology of the biopsy specimen (Hematoxylin and eosin stain) with intrafascicular tumour cells (asterisk) surrounded by collagen fibres (**a**). Epithelial membrane antigen staining is positive in tumour cells (asterisk) (**b**). S100 is not expressed in tumour cells but shows entrapped Schwann-cells in pseudo-onion bulbs (asterisk) (**c**). Immunohistochemical staining for Ki-67 reveals only very few proliferating tumour cells (asterisk) (**d**)

Histology of the biopsy specimen showed intrafascicular tumour cells surrounded by collagen fibres. Epithelial membrane antigen staining highlights concentric layers of tumour cells. S100 is not expressed in tumour cells but positive in the pre-existent Schwann cells. Immunohistochemical staining for Ki-67 reveals only very few proliferating tumour cells (Fig. 5). All cases showed a similar histomorphology and a comparable immunohistochemical expression profile, so that an intraneural PN was diagnosed in all cases.

### Results of the systematic review

The search resulted in 914 articles, after analysing the title and abstract 857 articles were excluded. The full text of the remaining 57 articles was reviewed and 35 articles were then excluded. Therefore, 22 articles met the inclusion criteria [5, 9, 12–31], comprising 74 cases of PN (Table 1).

**Table 1:** Results of the systematic review of the literature, including cases in which surgical treatment and follow-up is included

Year	Reference	Age (Years)	Sex	Nerve involved	Therapy	Follow-up	Outcome
1991	Phillips et al. [12]	Nm	male	ulnar	neurolysis complete resection + nerve graft	6 months	paralysis
1995	Emory et al. [5]	12	male	sciatic	fascicular biopsy	48 months	unchanged
		35	female	ulnar	fascicular biopsy	36 months	unchanged
		31	male	sciatic-tibial-branch	fascicular biopsy	17 months	unchanged
		15	male	median	fascicular biopsy	12 months	unchanged
		38	female	posterior interosseus	complete resection + nerve graft	3 months	worse
		18	female	femoral	complete resection + nerve graft	6 months	worse
		11	female	C8 & T1 nerve root	fascicular biopsy	1 months	unchanged
		13	female	peroneal	fascicular biopsy	3 months	unchanged
1998	Gruen et al. [13]	4	female	median	complete resection + nerve graft	5 years	improved
		14	female	peroneal	complete resection + nerve graft	2 years	little change
		8	male	peroneal	complete resection + nerve graft	2.5 years	foot brace
		4	male	median	complete resection + nerve graft	5 years	fair sensation, good thenar intrinsic motor function
		12	male	sciatic	internal neurolysis	4 years	Excellent pushdown, plantar sensation, foot brace
		38	female	median	internal neurolysis	6 years	reduced function
		21	female	brachial plexus	complete resection + nerve graft	6 years	unchanged
		16	female	peroneal	complete resection + nerve graft	4.25 years	improved
		37	male	ulnar	internal neurolysis	4 years	partial recovered
		37	male	radial	internal neurolysis	0,5 years	no follow-up
		16	male	median	complete resection + nerve graft	3 years	good recovery to date
		38	female	radial	complete resection + nerve graft	2 years	Good recovery to date
		8	female	peroneal	complete resection + nerve graft	0.5 years	none recovery
		43	female	peroneal	complete resection + nerve graft	1 year	early recovery
1999	Simmons et al. [14]	17	female	femoral	complete resection + nerve graft	10 months	unchanged
		12	female	sciatic	complete resection + nerve graft	2.5 years	unchanged
		26	female	brachial plexus	complete resection + nerve graft	2 months	unchanged
		19	male	Brachial plexus	neurolysis	6 months	improvement
2000	Jazayeri et al. [15]	53	male	Mmdian	complete resection + nerve graft	11 months	unchanged
2001	Alfonso et al. [16]	2	female	median	complete resection + nerve graft	12 months	unchanged
	Heilbrun et al. [17]	28	female	peroneal	dissection	5 years	unchanged
2004	Beekman et al. [18]	36	female	ulnar	neurolysis	6 months	improved
	Isaac et al. [19]	2	female	radial	Complete resection + nerve graft	3 years	unchanged
2005	Cortes et al. [20]	9	female	radial	complete resection + nerve graft	6 months	mild improvement
2007	Boyanton et al. [21]	5	male	ulnar	complete resection + nerve graft	3 years	some motor improvement
	Nguyen et al. [22]	30	female	radial	neurolysis, biopsy and tendon transfer	10 months	improved
2008	Gürkan et al. [23]	42	female	median	biopsy	16 months	improved
	Scheller et al. [24]	5	male	radial	complete resection + nerve graft	Postoperative Period	unchanged
		10	male	peroneal	complete resection + nerve graft	5 months	unchanged
		20	male	radial	complete resection + nerve graft	postoperative	first reinnervation
		29	male	ulnar	complete resection + nerve graft	postoperative	mild improvement
2009	Mauermann et al. [9]	12	female	sciatic	biopsy	5 months	unchanged
		7	malr	sciatic	biopsy	6 months	unchanged
		2	female	lumbosacral plexus	biopsy	18 months	Improvement in sensation
		12	female	sciatic	biopsy	78 months	unchanged
		35	male	tibial	biopsy	6 months	unchanged
		31	male	tibial	biopsy	177 months	improvement in stabbing sensation
		15	female	lumbosacral plexus	biopsy	88 months	mild increased weakness
		56	male	radial	biopsy	53 months	mild increased weakness
		19	female	femoral	biopsy	168 months	increased weakness
		7	female	sciatic	biopsy	118 months	unchanged
		34	male	sciatic	biopsy	45 months	increased numbness
		12	female	brachial plexus	biopsy	37 months	increased weakness
		35	male	brachial plexus	biopsy	36 months	worsened temperature discrimination
		30	female	radial	biopsy	27 months	improved in weakness and numbness
		14	male	sciatic	biopsy	24 months	increased atrophy
		30	male	sciatic	biopsy	18 months	unchanged
		12	male	brachial plexus	biopsy	34 months	increased atrophy
		8	male	sciatic	biopsy	135 months	improvement in weakness
		19	female	ulnar	biopsy	127 months	unchanged
		8	male	sciatic	biopsy	14 months	unchanged
		13	female	ulnar	biopsy	8 months	mild increased weakness
		37	male	tibial	biopsy	6 months	unchanged
	Sachanandani et al. [25]	23	female	median	fascicular biopsy	1 months	unchanged
2010	Ferraresi et al. [26]	4	male	peroneal	complete resection + nerve graft	7 years	unchanged
		9	male	sciatic	complete resection + nerve graft	8 years	unchanged
		13	female	peroneal	neurolysis	4 years	unchanged
		14	female	sciatic	neurolysis	5 years	unchanged
2011	Groeneweg [27]	22	male	ulnar	neurolysis + fascicular biopsy	nm	partial improvement
2012	Lavi et al. [28]	18	female	brachial plexus	biopsy + extzernal neurolysis	1 week	improved
2014	Chung et al. [29]	58	Female	brachial plexus	interfascicular dissection	18 months	nearly normal
2016	Dahlin et al. [30]	21	male	sciatic	fascicular biopsy	nm	unchanged
	McMillan et al. [31]	13	female	radial	complete resection + nerve graft	10 months	worse
		14	female	femoral	complete resection + end-t-side nerve transfer	18 months	mild improvement
	Case 1	56	male	median	fascicular biopsy + neurolysis interfascicular	27 months	improvement
	Case 2	16	male	ulnar	fascicular biopsy + neurolysis interfascicular	24 months	improvement
	Case 3	17	female	peroneal	fascicular biopsy + neurolysis interfascicular	30 months	improvement

Including our three cases, demographics, surgical treatment and the neurological outcome were analysed for 77 patients.

### Demographics

Mean age was 21.0 ± 13.9 years of age, 42 females (54.5%). Mean follow-up was 35.2 months. Sciatic nerve was affected mostly (*n* = 16, 20.8%), followed by peroneal (*n* = 11, 14.3%) and median nerve affection (n = 11, 14.3%).

### High-resolution nerve sonography

Typically, PN present as a fusiform enlargement of the nerve fascicles over several centimetres with hyperechoic perineurial tissue on HRNS. Fascicular structure of the enlarged nerves remains visible.

### Magnetic resonance imaging

Normally, PN show isointensity on T1-weighted and hyperintensity on T2-weighted MRI. Intraneural PN can show highly increased signal along the nerve on Short-Tau Inversion Recovery sequence with identifiable fascicles. Homogenous, moderate to strong contrast enhancement can be detected on T1-weighted contrast enhanced MRI.

### Surgery

Biopsy was performed in 32 patients (41.6%), complete resection and nerve grafting was performed in 28 cases (36.4%), whereas neurolysis (*n* = 12, 15.6%) or fascicular biopsy and interfascicular neurolysis (*n* = 5, 6.5%) was performed less.

### Neurological outcome

Neurological outcome was unchanged in 35 cases (45.5%), worsened in 16 cases (20.8%). Neurological improvement was detected in 26 patients (33.8%).

Analysis of the review of the literature revealed similar results between the different surgical treatment options. Complete resection with nerve grafting showed unchanged neurological outcome in 46.4% and an improvement in 35.7%. Single biopsy showed unchanged results of the neurological outcome in 53.1% and an improvement in18.8%. Unchanged neurological outcome was detected in 41.6% and improved neurological outcome was seen in 41.6% of the cases treated with neurolysis (Table 2).

**Table 2:** Different surgical therapy and its neurological outcome

Surgical Treatment	Neurological outcome		
	Worsened	Unchanged	Improved
Complete resection + nerve graft	5 (17.9%)	13 (46.4%)	10 (35.7%)
Biopsy	9 (28.1%)	17 (53.1%)	6 (18.8%)
Fascicular biopsy + neurolysis	0	0	5 (100%)
Neurolysis	2 (16.8%)	5 (41.6%)	5 (41.6%)

## Discussion

Intraneural PNs are rare benign peripheral nerve sheath tumours originating from perineurial cells, presenting slowly progressive, motor predominant focal neuropathy or plexopathy with mild sensory symptoms and signs [9].

Probably, intraneural PN is an under-diagnosed focal neuropathy, but more cases were detected over the last years due to a multidisciplinary approach by experts in peripheral nerve care, peripheral nerve imaging including HRNS, peripheral nerve surgery, electrophysiology and peripheral nerve pathology.

On MRI, PNs typically present as a fusiform enlargement of the nerve fascicles, isointense on T1-weighted and moderate to strong hyperintensity on T2-weighted MR images with moderate to strong homogenous contrast enhancement after intravenous gandolinium application [9, 32]. On HRNS examinations, a nerve enlargement over several centimetres with hyperechoic perineurial tissue can be detected. Fascicular structure of the enlarged nerves is still identifiable [10]. Up to date, HRNS is more and more commonly used for peripheral neuropathies [33], and is a well-established diagnostic tool, which can be used for follow-up examinations of known PNs. We believe that tumour growth can be detected easily with HRNS. Therefore, HRNS plays an important role in diagnosis and especially in follow-up examination in combination with the neurological examinations and the findings of the electrophysiological testing.

However, the treatment of intraneural PN is still a subject of great controversy despite several studies, mainly case reports, on this topic. A general consensus still does not exist. Surgery is commonly recommended in patients with a progressive neurological deficit and a localizable single lesion.

Gruen et al. recommended in their series of intraneural PNs that a surgical treatment should include resection of the lesion with interpositional nerve graft repair in cases where intraoperative nerve action potentials demonstrate a non-functioning or poorly functioning segment. Additionally, intraoperative histological examination is recommended to confirm onion- bulb neuropathy. They reported in their series, including 14 patients with localized hypertrophic neuropathy, the postoperative outcome after surgery. Seven out of 10 patients who received nerve grafting showed an improvement of the motor function. Four patients did not receive nerve grafting, but two of those patients recovered some function. Therefore, Gruen et al. concluded that routine nerve grafting should be performed [13].

Gruen and Kline also recommend that the lesion should be carefully resected until normal appearing fascicles and a bit further both proximally and distally of the lesion and nerve graft should be interposed, if no action potential or if poor amplitude was recorded across the lesion. More extensive nerve resection is made to avoid that the interpositioned nerve graft may eventually deteriorate as well, leading to a longer graft and therefore to poorer results [34]. In our opinion, extensive nerve resection and following nerve grafting has to be considered in patients with more distal located focal lesion without muscle atrophy. Otherwise, there is always the possibility of worsening disability or pain after surgical treatment of the nerve. Those criteria meet only in a few cases so that nerve resection should usually not be performed.

Heilbrun et al. reviewed the treatment options in patients with intraneural PN. They found 16 patients treated with resection of the tumour and nerve grafting. Collected follow-up information showed an improvement of motor function in eight cases and a slight improvement in two more patients. Three of five patients treated by tumour excision alone also demonstrated an improvement of the neurological deficits. They concluded that nerve grafting should be based on individually indications including negative intraoperative action potentials [17]. Early identification of the lesion and the treatment opportunity prior to irreversible deterioration of distal intramuscular nerve sheaths and longstanding denervation atrophy are also an important factor influencing the neurological outcome. Functional recovery after nerve grafting is most favourable in young patients [20].

Sachanandani et al. recommended as an alternative to proximal nerve grafting a distal nerve transfer in patients with progressive neuropathies due to several reasons. Widely resection of the lesion is possible using a nerve reconstruction that is distant to the lesion without any concern for the length of graft required [25]. Furthermore, reinnervation of the target musculature might be more successful to decreased length of regeneration required, elimination of an additional repair site, and exclusion of grafts [35]. Additionally, harvesting a nerve graft using a second surgery site and possible causing further deficits may be avoided [25].

Mauermann et al. present a dissenting opinion of the treatment of intraneural PN. In their opinion and current clinical practice, targeted fascicular nerve biopsy at the site of MRI lesion has to be performed as a much more focused nerve biopsy minimizing surgical deficits and postoperative scarring. Other surgical interventions such as tendon transfers or distal nerve grafting may be performed as a second stage procedure in patients there a static disease is evident after receiving the definitive diagnosis. Furthermore, clinically follow-up with imaging to verify clinical stability may be reasonable in patients there the nerve lesion is difficult to access without major invasive surgery [9].

We are in line with the therapeutically approach performing a targeted fascicular nerve biopsy at the site of MRI or HRNS lesion, but in our cases additionally interfascicular neurolysis, which is known to be a safe and reliable technique [36] had a positive impact on the neurological outcome.

Contrary to the common view and contrary to our approach, Restrepo et al. believe that a targeted fascicular biopsy is not necessary in patients there the clinical presentation (slowly progressive painless neuropathy and motor dysfunction with atrophy in the affected nerve territory) and the radiological imaging are typical for the presence of intraneural PN [37]. In our opinion, a targeted biopsy under electrophysiological monitoring is mandatory to receive a definitive diagnosis even in cases there the clinical presentation and the radiological imaging is highly suspicious of an intraneural PN to avoid unnecessary treatment.

However, neurological outcome did not differ between the analysed surgical treatment options (Table 2). Radiological evaluations of PN revealed more information about the course of this rare tumour. This might shift the surgical treatment from total resection and nerve grafting towards less invasive methods like targeted fascicular biopsy for diagnostic and wait-and-see strategies. This was suggested by Wilson et al., who were able to show that intraneural PN rarely growth in length and that they do not grow to involve new nerves or nerve divisions. Furthermore, growth does not correlate with clinical progression. Therefore, they concluded that intraneural PN have special characteristics, which can be detected radiological and which would make invasive diagnostic unnecessary [38].

Several limitations have to be addressed. First, this is a systematic review about a rare tumour. Therefore, we were only able to review case reports and small case studies. The informative value of those low evidence articles must be kept in mind. Secondly, neurological outcome was described only for several months.

However, future prospective studies with a longer follow-up are needed to evaluate the neurological long-term outcome in those patients.

## Conclusion

Intraneural PN is rare benign slowly progressive nerve sheath tumour. Treatment management is still controversy discussed but seems to become more and more less invasive. At least, targeted biopsy should be performed to receive histopathological diagnosis. HRNS is increasingly used for follow-up examinations.

## Data Availability

All data generated or analysed during this study are included in this published article.

## Abbreviations

HRNS: High resolution nerve sonography
MRI: Magnetic resonance imaging
PN: Perineurioma
